# Interfacial Engineering of Biocompatible Nanocapsules for Near‐Infrared‐Triggered Drug Release and Photothermal Therapy

**DOI:** 10.1002/advs.202410844

**Published:** 2024-11-21

**Authors:** Yuting Xie, Ze Yang, Hang Shen, Jingyi Chen, David A. Weitz, Dong Chen, Jianpeng Sheng, Tingbo Liang

**Affiliations:** ^1^ Department of Hepatobiliary and Pancreatic Surgery The First Affiliated Hospital Zhejiang University School of Medicine Hangzhou 310003 China; ^2^ Zhejiang Provincial Key Laboratory of Pancreatic Disease The First Affiliated Hospital Zhejiang University School of Medicine Hangzhou 310003 China; ^3^ Department of Medical Oncology The First Affiliated Hospital School of Medicine Zhejiang University Hangzhou 310003 China; ^4^ College of Energy Engineering and State Key Laboratory of Clean Energy Utilization Zhejiang University Hangzhou 310003 China; ^5^ John A. Paulson School of Engineering and Applied Sciences Harvard University Cambridge MA 02138 USA

**Keywords:** controlled release, interfacial engineering, nanocapsule, near‐infrared, phototherapy

## Abstract

Chemotherapy is an effective option for cancer treatment. However, its clinical application is often limited by the severe side effects of chemical drugs. To overcome these limitations, a novel drug‐loaded phase‐change nanocapsule system is developed. These nanocapsules are assembled via one‐step electrostatic self‐assembly through guided interfacial engineering. The phase change material core nanocapsules demonstrate great photothermal‐controlled drug release performance and exhibit excellent tumor‐targeting drug delivery performance both in vitro and in vivo via the binding of hyaluronic acid shell on the nanocapsule surface with corresponding receptors on the tumor cell membrane. The phototherapy function of the nanocapsules enhances immune activation within the tumor microenvironment, as demonstrated by flow cytometry and multiplex immunohistochemistry. The developed nanocapsules are biocompatible, versatile, and scalable and offer a promising smart delivery platform for controllable near‐infrared triggered drug release and photothermal therapy.

## Graphical abstract

Biocompatible nanocapsules are prepared by encapsulating natural phase change material in a hyaluronic acid shell via electrostatic interfacial engineering. The nanocarrier can controllably release the payload drug under irradiation. The smart delivery nanocarrier is a potential approach for tumor‐targeting drug delivery and reduces the side‐effect meanwhile the photothermal therapy is synergic to the chemotherapy.

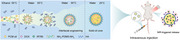

## 1 Introduction

Chemotherapy is the most commonly used cancer treatment approach for delivering chemical drugs to tumors.^[^ ^1^ ^]^ However, the toxic side effects of current chemotherapeutic agents, such as blood disorders and cardiac toxicity, limit their clinical applicability and pose significant risks to patients.^[^ ^2^ ^]^

Smart delivery technology is a potential approach to solve the problem owing to its controlled release function only under different stimuli, such as pH,^[^ ^3^ ^]^ temperature,^[^ ^4^ ^]^ ultrasound,^[^ ^5^ ^]^ specific biomolecule,^[^ ^6^ ^]^ and light.^[^ ^7^ ^]^ Among this tech, light offers the advantages of harmless noninvasiveness, simple controllability, and high resolution, and thus ithas the potential to build an effective smart delivery platform.^[^ ^8^ ^]^ In general, light‐responsive delivery platforms are usually achieved by breaking the thermosensitive polymer to release the drug payload with a photothermal effect induced by the photosensitizer. However, challenges remain in translating these synthetic polymer systems into clinical use due to their complex fabrication processes, limited degradability, and potential cytotoxicity.^[^ ^9^ ^]^ Therefore, in recent years, light‐responsive nanocarriers have been developed by utilizing phase change materials as a thermal‐responsive platform, since the payload trapped in the solid phase change materials can be released in response to a photothermal effect due to their reversible solid‐liquid transition characteristic over a narrow temperature range.^[^ ^10^ ^]^

Natural fatty acids are favored phase change materials due to their low cost, chemical stability, and biocompatibility.^[^ ^11^ ^]^ However, nanocarriers made of fatty acids usually have poor dispersibility in an aqueous medium. They tend to aggregate into larger particles and then float on the surface of an aqueous solution.^[^ ^12^ ^]^ It is challenging to prevent the fatty acids from pre‐degradation in vivo and the payloads from pre‐leakage.

Therefore, in order to deliver this phase change material to the tumor, nanocarriers, such as lipid nanocapsules^[^ ^13^ ^]^ and polymer nanocapsules^[^ ^14^ ^]^ are developed to encapsulate phase change materials to avoid them degrading in vivo. However, the poor dispersibility and colloidal stability of such nanocarriers under physiological conditions place a major limitation on their applications in biomedicine. In addition, the complex preparation methods and low encapsulation efficiency of these nanocarriers present further obstacles.^[^ ^15^ ^]^ There is a critical need for simpler and more efficient methods to design nanocarriers that can precisely deliver phase change materials for controlled drug release.

**Figure 1:**
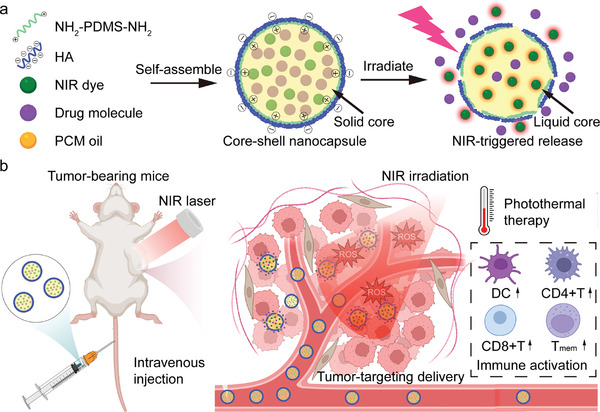
Interfacial engineering of biocompatible core‐shell nanocapsules for near‐infrared‐triggered drug release and photothermal therapy. a) Preparation of drug‐loaded nanocapsules via guided interfacial electrostatic self‐assembly. The solid core, composed of a eutectic mixture of two fatty acids, acts as a phase change material, while a near‐infrared laser facilitates drug release through the photothermal effect of a NIR dye. b) Tumor‐targeting drug delivery and controlled drug release of nanocapsules in vivo. The immune activation effect within the tumor microenvironment is attributed to the photothermal therapy under NIR irradiation.

In this work, a green and versatile strategy is developed to prepare biocompatible core‐shell nanocapsules in a single step. Instead of using poisonous cationic surfactants, a biocompatible positive amphiphilic polymer is introduced as a linkage to encapsulate phase change oil into a hyaluronic acid shell. Upon rapid mixing and solvent exchange, drug, photosensitizer, and phase change oil self‐assemble into an oil droplet with amphiphilic polymer located on the surface, and then HA in water coats the oil droplet because of the electrostatic interaction. The solid core of nanocapsules will turn into a liquid phase under near‐infrared (NIR) irradiation and release drugs (**Figure** **1**a). The prepared core‐shell nanocapsules are well dispersed in water and show great thermal performance and excellent NIR‐triggered release performance. Nanocapsules with tumor‐targeting function show excellent anti‐tumor performances both in vitro and in vivo. In addition, flow cytometry and multiplex immunohistochemistry staining reveal the immune activation in the subcutaneous and orthotopic tumor microenvironment which is attributed to photothermal therapy under NIR irradiation (Figure 1b). All the results suggest that the biocompatible core‐shell developed in this work are promising smart nanocarriers for near‐infrared‐triggered drug release and photothermal therapy.

## 2 Result

**Figure 2:**
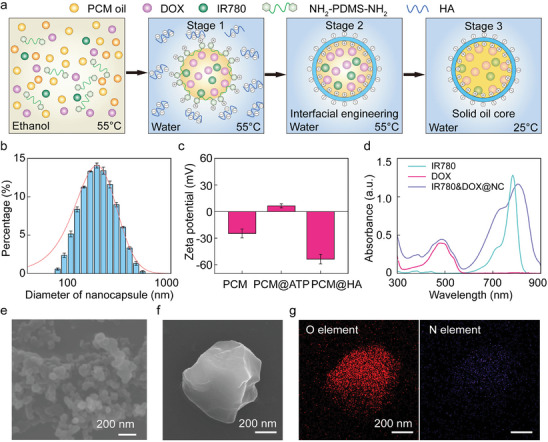
Characterization of nanocapsules prepared by electrostatic self‐assembly. a) Schematic illustration of interfacial electrostatic self‐assembly of core‐shell nanocapsule upon rapid solvent exchange in three stages: i) small oil molecules aggregate to form oil nanodroplets first and amphiphilic NH2‐PDMS‐NH2 molecules preferentially gather at the oil/water interface to form positive oil droplet, ii) Negative HA molecules self‐assemble at the droplet surface due to electrostatic interaction to form core‐shell nanocapsule. iii) the oil core of the nanocapsule becomes solid while the room temperature is lower than the melting temperature of the eutectic mixture. b) Size distribution of nanocapsules measured by DLS. c) Zeta potential of unencapsulated PCM, PCM mixes with NH2‐PDMS‐NH2 (PCM@ATP), and PCM encapsulated in nanocapsules (PCM@HA) at 55 °C d) UV–vis absorption spectra of unencapsulated DOX, unencapsulated IR780, and IR780+DOX‐loaded nanocapsules. e)SEM image of nanocapsules. f)SEM image and g) EDS image of an empty nanocapsule after removal of the oil core.

Instead of using unstable lipid material to encapsulate the phase change oil core, a negative amphiphilic biocompatible polymer α, ω‐diamino functionalized polydimethylsiloxane (NH_2_‐PDMS‐NH_2_, Amino‐terminated polydimethylsiloxane, ATP), which consists of one hydrophobic PDMS backbone and two hydrophilic amino end groups, is used as the tool material to attract hyaluronic acid (HA) to coat the surface of phase change oil core as a shell.^[^ ^16^ ^]^ In this study, doxorubicin (DOX) and IR‐780 iodide (IR780) are used as the model lipophilic drug and NIR dye. The primary advantage of our IR780+DOX photothermal release model lies in its targeted release mechanism, which ensures that DOX is released specifically at the tumor site. The critical temperature of the phase change material we used is close to body temperature at 39 °C.^[^ ^17^ ^]^ The photothermal therapy we applied reaches this temperature without damaging normal tissues. To self‐assemble the nanocapsule, a mixture of LA (Lauric acid) and SA (Stearic acid), IR780, DOX, and NH_2_‐PDMS‐NH_2_ is co‐dissolved in ethanol at 55° C, while HA is dissolved in water at 55° C. The nanocapsules are prepared by rapid solvent exchange and interfacial electrostatic self‐assembly. During the rapid mixing process, the solvent mixing time (t_mix_ ≈ 10 ms) is smaller than the molecule aggregation time (t_agg_ ≈ 30 ms).^[^ ^18^ ^]^ Therefore, solvent exchange is completed before molecule aggregation and the self‐assembly of nanocapsules undergoes three stages, as schematically shown in **Figure** **2** a: i) small oil molecules aggregate to form oil droplets first with DOX and IR780 preferably dissolved in them due to their lipophilic characteristic; ii) NH_2_‐PDMS‐NH_2_ preferentially gather at the oil/water interface due to its amphiphilic characteristic; iii) negative HA self‐assembles on the droplet surface to form the nanocapsules. The sample will then cool down in an ice bath to solidify the phase change material (PCM) oil core. The nanocapsules are uniform sizes analyzed by DLS (d ≈ 183 ± 44 nm) as shown in Figure 2b. The loading of DOX and IR780 in the nanocapsule does not affect the characteristics of nanocapsules as shown in Table S1 (Supporting Information). In addition, zeta potential is used to prove the PCM oil core is encapsulated by HA via electrostatic attraction, as shown in Figure 2c, while the oil droplet shows positive with the presence of NH_2_‐PDMS‐NH_2_ and becomes negative after being encapsulated by negative HA molecules, which is attributed to the electrostatic interaction between NH_2_‐PDMS‐NH_2_ and HA. IR780 and DOX are proved to be encapsulated in nanocapsules by UV‐vis as shown in Figure 2d. The encapsulation efficiency and loading capacity of IR780 and DOX in nanocapsule (IR780+DOX@NC) is estimated by UV–vis as shown in Table S2 and Figures S1–S4 (Supporting Information). The great encapsulation efficiency is attributed to the presence of the oil core while all the payload are lipophilic. It is worth noting that after encapsulation, the absorption peak of IR780 is redshifted by 10 nm from 780 to 790 nm, together with broadening, which is due to the hydrophobic interaction between IR780 and the PCM matrix.^[^ ^19^ ^]^ The morphology of nanocapsules is directly observed by scanning electron microscopy (SEM) in Figure 2e, which is represented as solid core nanocapsule under room temperature. After the removal of the oil core, the nanocapsule shows cramped shell morphology as shown in Figure 2f, which supports the core‐shell structure of nanocapsules. The distribution of slight nitrogen and oxygen elements in the energy dispersive spectrometer (EDS) image in Figure 2g also confirms the core–shell structure and the shell is assembled by HA and NH_2_‐PDMS‐NH_2_.

**Figure 3:**
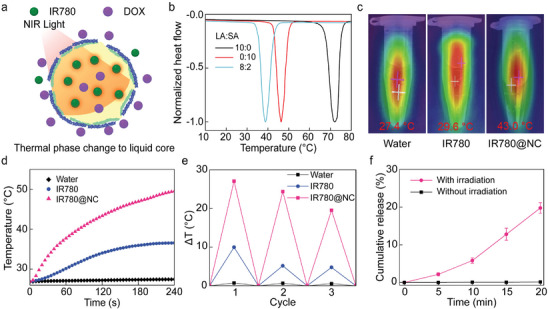
Photothermal performance of nanocapsules. a) Scheme illustration of NIR‐triggered release of nanocapsule. The thermal phase change is induced by the photothermal effect of IR780 under NIR irradiation. b) Differential scanning calorimetry curves of lauric acid (LA) and stearic acid (SA) at various weight ratios. Different mixing ratios of LA and SA show different melting points. c) Infrared image of pure water, unencapsulated IR780 and IR780@NC dispersion in water under NIR irradiation (0.6 W cm^−2^). d) Temperature changes unencapsulated IR780 and IR780@NC dispersion in water under a NIR irradiation NIR laser (0.6 W cm^−2^). Pure water under laser irradiation is used as a control. e) Comparison of the photothermal heating behaviors between unencapsulated IR780 and IR780@NC dispersion in water after repeated cycles of laser irradiation. f) Cumulative DOX release from the IR780+DOX@NC nanocapsules under a NIR irradiation NIR laser (0.6 W cm^−2^).

To meet the smart controlled release demand, the photosensitizer IR780 is incorporated inside the PCM oil core to induce a photothermal effect as shown in **Figure** **3**a. Under NIR irradiation, the solid oil core will melt into a liquid core due to the thermal effect. In this study, a eutectic mixture of lauric acid and stearic acid (4:1 wt%) is used as a phase change oil core, which exhibits a sharp melting point of 39 °C as shown in Figure 3b. As naturally occurring fatty acids, both lauric acid and stearic acid are biocompatible and biodegradable.^[^ ^20^ ^]^ Compared to many other thermal change fatty acids, these two biocompatible natural fatty acids have favorable effects on decreasing atherosclerotic risk.^[^ ^21^ ^]^

To evaluate the photothermal properties of IR780‐loaded nanocapsules, an 808 nm NIR laser is introduced to excite the IR780. The saturated water solution of free IR780 and dispersion of IR780 nanocapsules in water are irradiated by 808 nm NIR laser (0.6 W cm^−2^). The thermal effect is not well dispersed in water as free IR780 as shown in Figure 3c. This is because IR780 is insoluble in water and floats on top of water. The photothermal performance of IR780@NC is greater than unencapsulated IR780 as monitored in Figure 3d. The great photothermal properties of nanocapsules could contribute to the separation of IR780 by nanocapsule shell and solid PCM core, which reduces the aggregation and degradation of IR780. The stability of IR780 is greatly enhanced after encapsulating in nanocapsules compared to free IR780 dispersed in water after placing in the open for 3h as shown in Figure S5 (Supporting Information). In addition, the performance is proved again by repeated laser irradiation (5 min for each round and separated by 10 min), while the IR780 nanocapsules show a potential repeatedly triggered release performance, which may be beneficial to controlled specific release as shown in Figure 3e. The release profile of IR780+DOX@NC is investigated with and without irradiation, nearly twenty percent of DOX is released under NIR laser (0.6 W cm^−2^) for 20 min as shown in Figure 3f and Table S3 (Supporting Information). This controlled release behavior highlights the potential for further clinical applications of IR780‐loaded nanocapsules.

**Figure 4:**
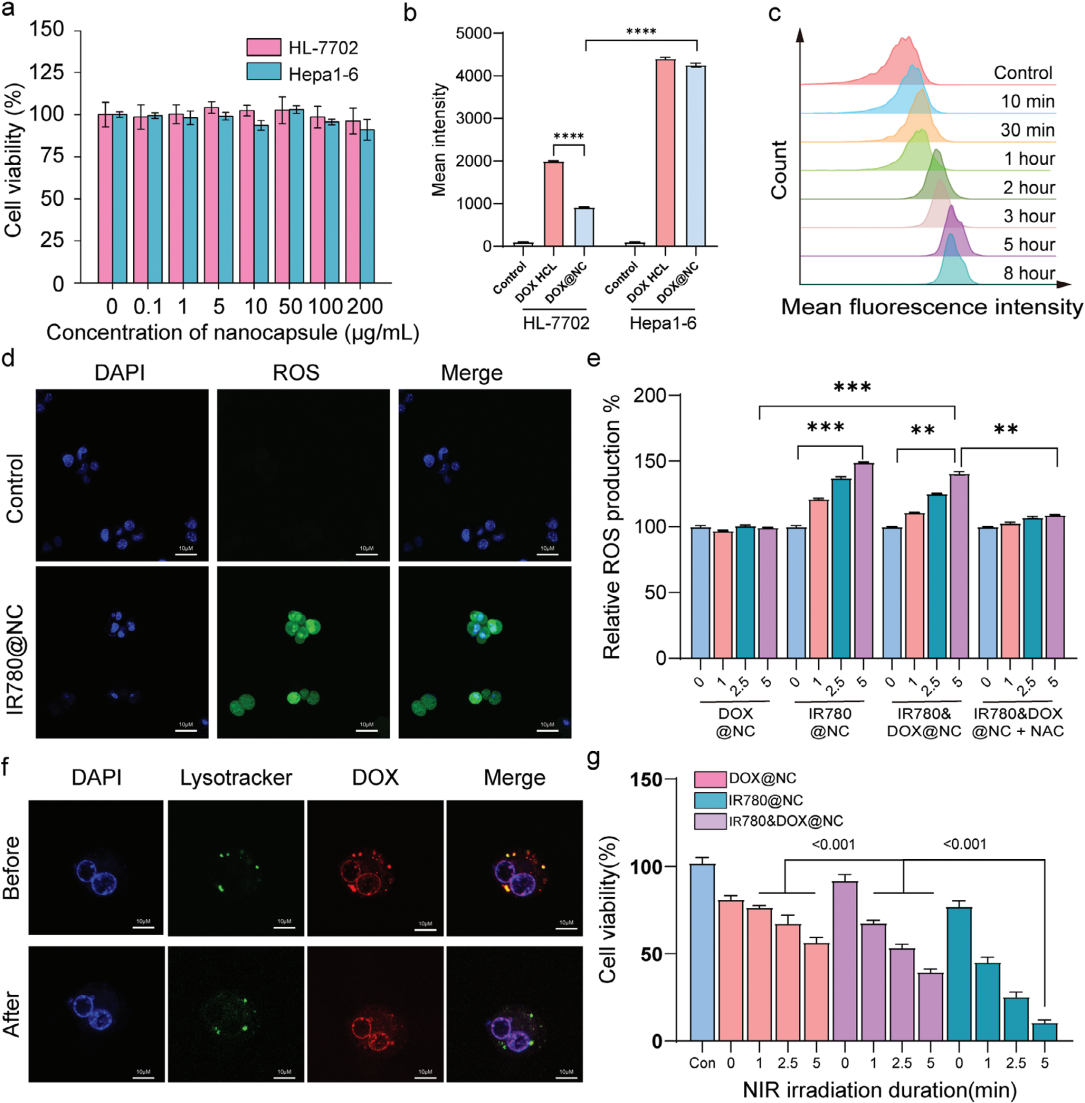
Biocompatibility and bioavailability of nanocapsules in vitro. a) Cell viability of Hpea 1–6 and HL‐7702 cells exposed to blank nanocapsules of different concentrations. b) Mean fluorescent intensity of Hpea 1–6 and HL‐7702 cells after 5 h of exposure to unencapsulated DOX and DOX@NC as measured by flow cytometry. c) Pharmacokinetics analysis of DOX@NC in Hepa 1–6. d) Fluorescent image of intracellular ROS production in Hepa 1–6 cells treated with IR780@NC nanocapsules under NIR irradiation. e) Relative rate of intracellular ROS production in Hepa 1–6 cells treated with DOX@NC, IR780@NC, IR780+DOX@NC and IR780+DOX@NC+NAC under NIR irradiation (0.6 W cm^−2^). f) Cellular distribution of DOX under irradiation. Fluorescence images showing the locations of nuclear, lysotracker, and DOX in Heap 1–6 cells pre and post NIR irradiation (0.6 W cm^−2^). g) Cell viability of Hepa 1–6 cells incubated of DOX@NC, IR780@NC, and IR780+DOX@NC under laser irradiation (0.6W cm^−2^) for a different duration. The IR780 concentration was 0.5µg mL^−1^ and the DOX concentration was 1µg mL^−1^. Samples for each condition (n = 3) were averaged and normalized to the cell number (5000).

Since no toxic solvents are used and all oils and polymers are biocompatible, the core‐shell nanocapsules without a payload exhibit excellent biocompatibility as demonstrated in the cell culture experiments with Hepa1‐6 and HL‐7702 cells, as shown in **Figure** **4**a. Doxorubicin Hydrochloride (DOX‐HCl) is a common clinical chemical drug and has been used to treat a variety of tumors. However, the application of water‐soluble DOX‐HCl is limited by its side effects such as cardiotoxicity, hepatic toxicity, or renal toxicity.^[^ ^22^ ^]^ Therefore, we chose a desalted modification to obtain a lipophilic form of DOX, combined with the controlled release design of our material, this can significantly reduce side effects associated with DOX‐HCl. The uptake results of HL‐7702 and Hepa 1–6 cells introduced into the culture with DOX‐HCl and DOX@NC for 5h are measured by flow cytometry as shown in Figure 4b, the nanocapsules possess a great tumor targeting ability that shows a much higher fluorescent intensity in Hepa 1–6 cells and greater selectivity to with show a lower fluorescent intensity compared to free DOX‐HCL in HL‐7702 cells. While the nanocapsules are taken up by endocytosis instead of diffusion, the nanocapsule shows greater selectivity than the DOX molecule attributed to the specific target ability to CD44‐overexpressing tumor cells via HA.^[^ ^23^ ^]^ To investigate the pharmacokinetics of nanocapsules in vitro, a quantitative analysis of labeling efficiency is performed by flow cytometry as shown in Figure 4c, the results reveal that DOX encapsulated in nanocapsules show a great resistance of clearance compared to free DOX and cumulate highest concentration of DOX in cells at 5 h.^[^ ^24^ ^]^ In addition, the IR780 can sensitize the production of ROS under the irradiation of NIR light,^[^ ^25^ ^]^ which is confirmed by the fluorescence image in Figure 4d, while the ROS is stained by the DCHF‐DA probe. The existence of IR780 greatly enhances ROS production in cellular. The ROS production of Hepa 1–6 cells exposed to DOX@NC, IR780@NC, IR780+DOX@NC and IR780+DOX@NC + NAC (N‐acetyl‐L‐cysteine, a ROS scavenger) is investigated in Figure 4e, in which the loaded of IR780 is confirmed to produce a certain amount of ROS in cells after irradiation, and increase of ROS can be reversed by NAC. Intracellular localization of DOX in Hepa 1–6 cells is investigated after co‐incubation of the IR780+DOX@NC for 5 h, which shows a highly co‐localized fluorescence of DOX and lysotracker Figure 4f, which suggests that the IR780+DOX@NC nanocapsule entered cells via an endolysosomal pathway. Under irradiation, the increase of temperature in cellular above the melting point of PCM may quickly trigger intracellular release of the encapsulated from the nanocapsules, which also benefits from the non‐dense coating structure of HA on the surface of nanocapsules. The escape of DOX from endolysosome is observed under NIR irradiation which is attributed to the photothermal effect of IR780, while DOX does not release through endolysosome as shown in Figure S6a (Supporting Information). The intracellular controlled release process is also monitored in Figure S6b (Supporting Information). After irradiation with the NIR laser for 2.5 min, DOX starts to move from the acidic compartments to the cytosol, as characterized by the blurred and diffusive fluorescence. Meanwhile, some DOX is found to accumulate in the periphery of the nucleus. At 5 min post‐irradiation, the distribution of DOX in the cytosol became more homogenous, with a barely recognized punctate pattern. More importantly, the nuclear shape is clearly outlined by the fluorescence of DOX. The 10 min fluorescence image indicates that a large amount of DOX escaped from the endolysosomes and intercalated into the nuclear DNA. It should be pointed out that since the internalized nanocapsules are primarily localized in the endolysosomes, drugs cannot escape from these acidic compartments and diffuse into the cytosol. In this process, ROS production also gives rise to the destabilization of the endolysosomal membranes to facilitate cytosolic diffusion of the drug. These findings suggest that NIR irradiation not only initiates the release of DOX from the nanocapsules but also facilitates its escape from endolysosomes into the cytosol, contributing to effective drug delivery.^[^ ^26^ ^]^

**Figure 5:**
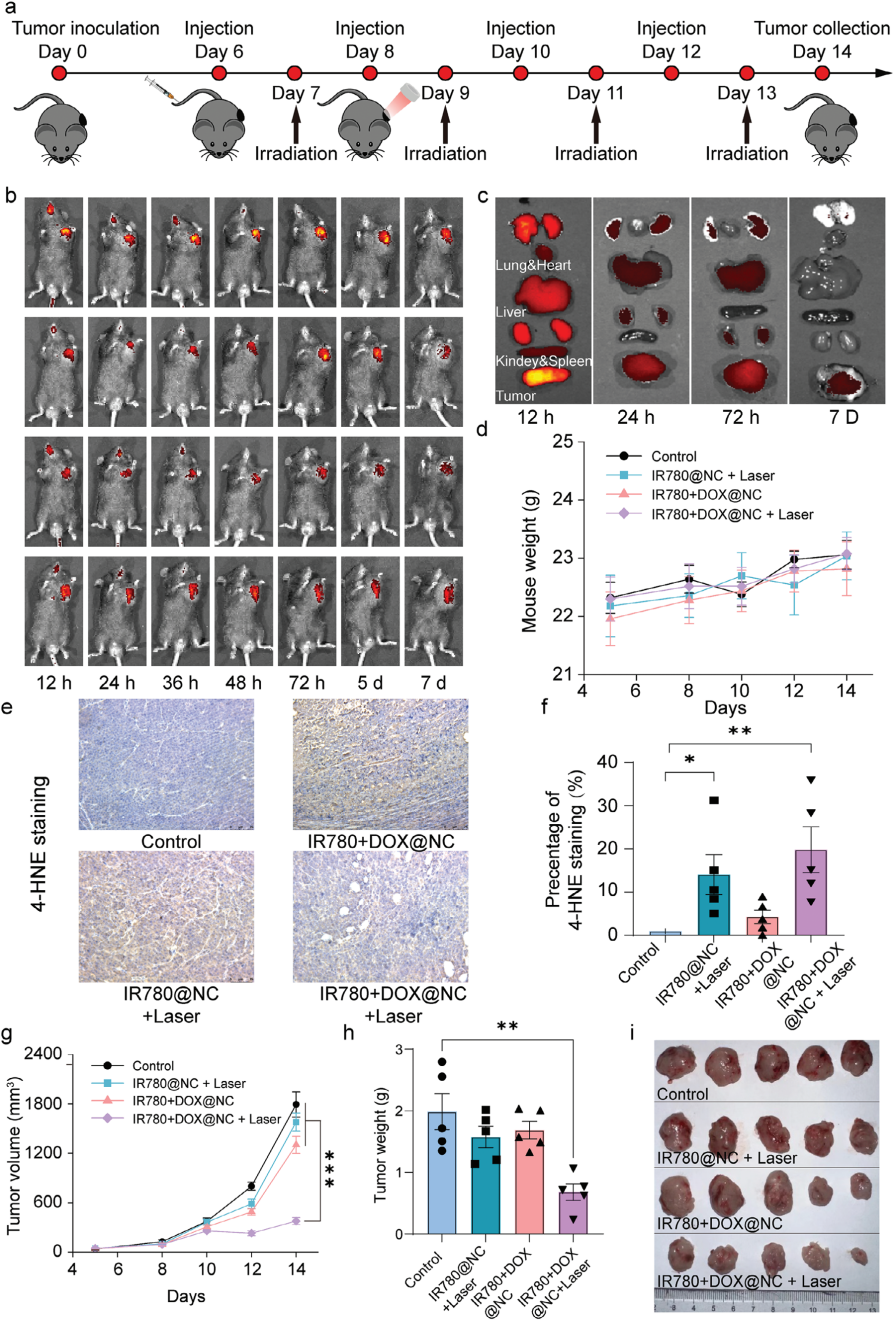
Anti‐tumor performances of drug‐loaded nanocapsules in vivo. a) Schedule of tumor‐bearing mice therapy with nanocapsules and irradiation. Tumor‐bearing mice were i.v. Injected drug‐loaded nanocapsules (DOX 1 mg kg^−1^, IR780 0.5 mg kg^−1^) or control formulations. b) In vivo imaging of mice after just a single intravenous injection of IR780+DOX@NC for 7 days. c) Fluorescence image of organ dissected from mice after intravenous injection of IR780+DOX@NC d) Weight of tumor‐bearing mice under different treatments over time. e) Immunohistochemical staining and f) quantification of 4‐HNE in tumor. g) The volume of tumors in tumor‐bearing mice under different treatments over time. h) Weight of tumors dissected from mice after two weeks’ treatments. i) Photograph of tumors dissected from mice after two weeks’ treatments.

The promising in vitro activity of IR780+DOX@NC+laser has prompted us to investigate its therapeutic efficacy in vivo. We first assessed its pharmacokinetics and biodistribution profile using the intrinsic fluorescence signal of IR780. IR780+DOX@NC demonstrated prolonged circulation in **Figure** **5**b. Consistent with the pharmacokinetics, IR780+DOX@NC and IR780@NC exhibited enhanced permeability and retention (EPR) effects, resulting in increased accumulation in the tumor site.^[^ ^27^ ^]^ Notably, IR780+DOX@NC showed stable accumulation from 12 h to 7 days post intravenous injection in Figure 5b, while IR780@NC also shows a great tumor‐targeting performance in Figure S7 (Supporting Information). Fluorescent images of dissected organs further confirm the accumulation of nanocapsules at the tumor site and thus the tumor‐targeting performance, as shown in Figure 5c, while the main part of nanocapsules is cleaned by other organs at 7 days. This is because the expression of CD44 receptors is significantly higher in cancers than in normal tissues and nanocapsules can effectively bind to the cancer cells via the binding of HA with CD44 receptor.^[^ ^28^ ^]^ The result is consistent with the uptake result in vitro. The tumor‐targeting capability is essential for in vivo drug delivery, which could improve the anti‐tumor performance and reduce the side effects.

Tumor‐bearing mice are treated with drug‐loaded nanocapsules by intravenous injection every 3 days for 10 days, and one day after the injection, each mouse is irradiated by an 808 nm NIR laser (0.8 W m^−2^) for 20 min, as shown in Figure 5a. After two weeks’ treatments with PBS, IR780@NC, and IR780+DOX@NC, no significant weight loss is observed in the four groups, suggesting that nanocapsules are well tolerated by mice, as shown in Figure 5d. Following laser treatment, the peroxidative damage byproduct 4‐hydroxy‐2‐nonenal (4‐HNE) in the tumor is upregulated as shown in Figure 5e, suggesting that the laser induces reactive oxygen species, and the IR780+DOX@NC with irradiation group induces the most oxidative stress in the tumor as resulted in Figure 5f. As shown in Figure 5g, without NIR light, only a slightly therapeutic effect is observed after the injection, indicative of little toxicity of nanocapsules. However, the group treated with IR780+DOX@NC with irradiation shows great performance in suppressing the tumor growth in vivo under irradiation as shown in Figure 5h, which proves the controlled release characteristic of nanocapsule in vivo. This group shows a significantly smaller tumor volume and weight compared with the group without irradiation. The tumor in Figure 5i further confirms the great therapy performance of IR780+DOX@NC nanocapsules under NIR irradiation. The result proves that the better anti‐tumor effect is carried out by the synergistic performance of controlled release of the DOX and photothermal therapy of IR780 rather than only inducing ROS in the tumor microenvironment (TME) via photosensitizer under irradiation. Compared to mice that have been treated with conventional chemotherapy, our nanocapsule platform shows a greater tumor inhibition performance.^[^ ^29^ ^]^

To evaluate the side effects of drug‐loaded nanocapsules, the inflammation responses of the five major organs, i.e. heart, liver, spleen, lung, and kidney of mice under different treatments, are analyzed by hematoxylin and eosin staining (H&E staining), as shown in Figure S8 (Supporting Information). No distinguishable differences are observed between the five major organs of mice under different treatments, suggesting negligible toxicity. The minimized drug toxicity and side effects of drug‐loaded nanocapsules are attributed to their excellent biocompatibility, controlled release characteristics, and tumor‐targeting capability.

**Figure 6:**
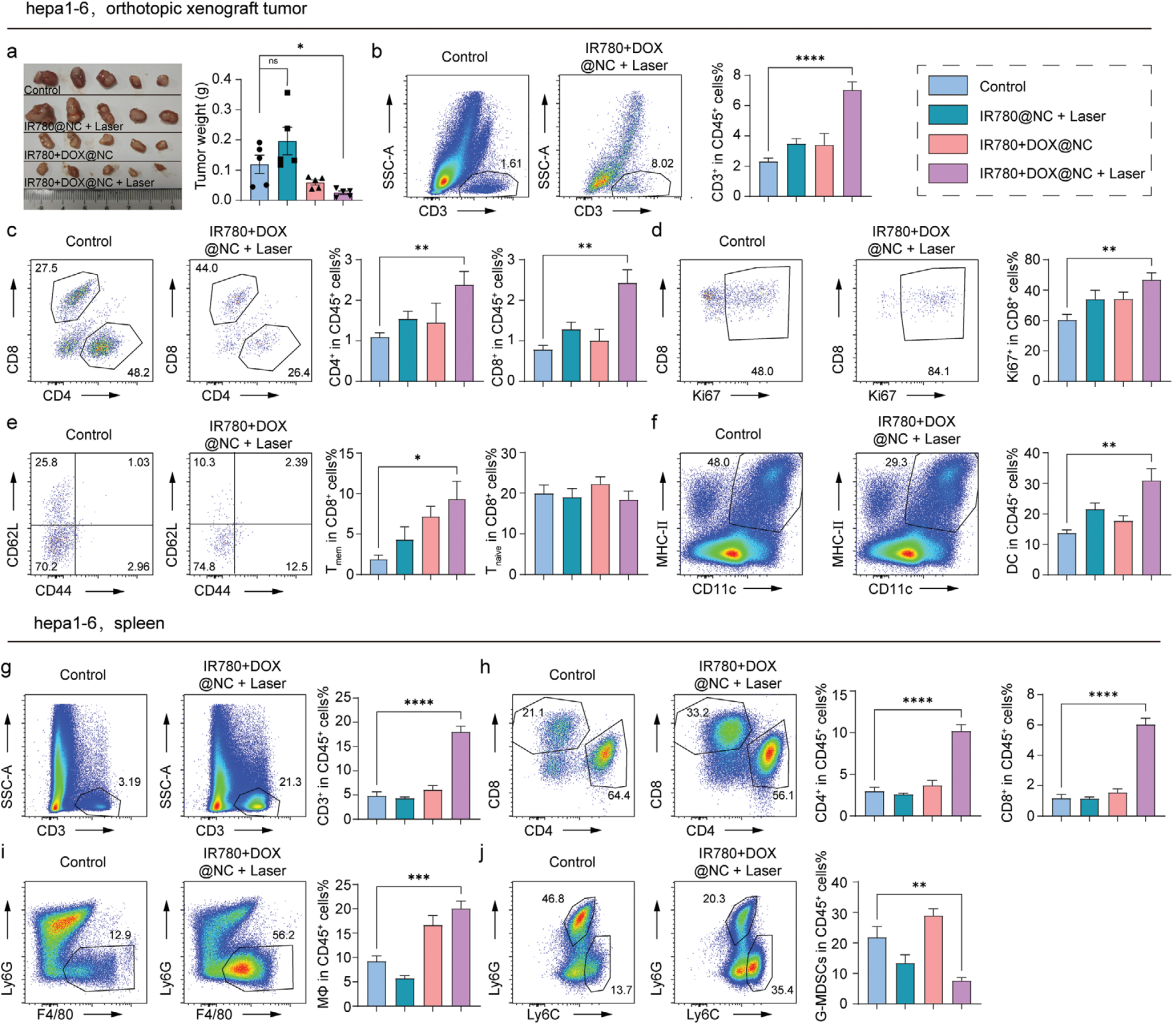
Immune remodeling of tumor microenvironment by nanocapsules in vivo. a)Photograph of orthotopic tumors dissected from mice after two weeks’ treatments. Ratios of b) CD3^+^ T, c) CD4^+^ T, CD8^+^ T cells, d) Ki67^+^, e) Memory T (T_mem_), Naïve T (T_Naive_), and f) dendritic cells (DCs) in the orthotopic tumor microenvironments of mice under different treatments. Ratio of g) CD3^+^ T, h) CD4^+^ T, CD8^+^ T cells, i) macrophage cells (MΦ), and j) granulocytic myeloid‐derived suppressor cells (G‐MDSCs) in spleens.

The photothermal effect and ROS production of photosensitizer under NIR irradiation will also induce antitumor strong immunity and remodel the tumor microenvironment.^[^ ^30^ ^]^ To assess whether local chemotherapy and photothermal therapy treatment can induce an antitumor immune response to effectively treat distant tumors. A subcutaneous Hepa1‐6 tumor model is established with an orthotopic xenograft in the liver to evaluate the distant therapeutic effects. Animals were treated as described above. The results are promising, while the therapy effectively reduced distant tumor burden, as shown in **Figure** **6**a, indicating the potential induction of systemic immune responses which is investigated by flow cytometry as shown in Figure S9 (Supporting Information). However, mice treated with IR780+DOX@NC without irradiation exhibit limited distant effects. To further explore the high activity of phototherapy‐mediated treatment, the immune response is examined, as shown in Figure 6b–f. Immune profiling of the tumors after treatment reveals the induction of robust dendritic cells (DCs), and CD4^+^ T cell increase to present tumor antigen and recruitment of tumor‐infiltrating CD8^+^ T cells to kill tumor cells, while with a significant upregulation of Ki67 in CD8^+^ T cells, indicating enhanced proliferation. Higher frequencies of Memory T (T_mem_) cells are observed in the CTL populations of IR780+DOX@NC with irradiation group compared to other groups, while the proportion of naïve T cells (T_Naive_) shows no significant difference among groups, indicating a crucial characterization for distal tumor inhibition. To provide a clear illustration of the immune reaction at a distance, flow cytometry is used to qualify the immune response in the spleen. Analysis indicates that the IR780+DOX@NC+Laser treatment group promoted the infiltration of quantified CD3^+^ T cells, CD8^+^ T cells, CD4^+^ T cells, and macrophage cells (MΦ) while reducing the infiltration of granulocytic myeloid‐derived suppressor cells (G‐MDSCs), which suppress the immune response, compared to other groups, as shown in Figure 6g–j. These results demonstrate significant immune activation in vivo through chemotherapy and photothermal therapy, further supporting the excellent antitumor performance of the nanocapsules.

## 3 Conclusion

In this study, a versatile nanocapsule with a phase change material core is developed through one‐step interfacial electrostatic engineering. A eutectic mixture of natural fatty acids is utilized as a biocompatible phase change oil core, enabling controlled drug release under NIR irradiation for precise drug delivery. In addition, the photothermal effect triggered by NIR significantly boosts ROS production, further enhancing the cytotoxic effects on tumors. We believe that utilizing minimally invasive endoscopy for this drug delivery method is an effective application strategy, and we are continuously working to improve this approach for better clinical implementation. The developed nanocapsules exhibit remarkable synergetic effects in drug release and photothermal therapy, demonstrating high bioavailability, low side effects, and enhanced anti‐cancer activity through their combined benefits. This excellent nanocarrier platform shows broad application potential, offering an innovative solution for precise drug delivery and tumor treatment.

## Conflict of Interest

The authors declare no conflict of interest.

## Supporting information



Supporting Information

## Data Availability

The data that support the findings of this study are available from the corresponding author upon reasonable request.
