# Supplementary material for: Interfacial Engineering of Biocompatible Nanocapsules for Near‐Infrared‐Triggered Drug Release and Photothermal Therapy
Source: Adv Sci (Weinh). 2024 Nov 21;12(2):2410844. doi: 10.1002/advs.202410844 (PMC11727245; doi:10.1002/advs.202410844)
Supplement: Supplementary file 1 — Supporting Information [file ADVS-12-2410844-s001.docx]

Supporting information

**Interfacial Engineering of Biocompatible Nanocapsules for Near-Infrared-Triggered Drug Release and Photothermal Therapy**

*Yuting Xie^+^, Ze Yang^+^, Hang Shen^+^, Jingyi Chen, David A Weitz, Dong Chen*, Jianpeng Sheng* and Tingbo Liang**

^+^These authors contribute equally to this work

Y. Xie, H. Shen, Prof. J. Sheng, Prof. T. Liang

Department of Hepatobiliary and Pancreatic Surgery, The First Affiliated Hospital, School of Medicine, Zhejiang University, Hangzhou 310003, China

E-mail: shengjp@zju.edu.cn

E-mail: [liangtingbo@zju.edu.cn](mailto:liangtingbo@zju.edu.cn)

Z. Yang, Prof. D. Chen

Department of Medical Oncology, The First Affiliated Hospital, School of Medicine, Zhejiang University, Hangzhou 310003, China

E-mail: [chen_dong@zju.edu.cn](mailto:chen_dong@zju.edu.cn)

Z. Yang, Prof. D. Chen

College of Energy Engineering and State Key Laboratory of Clean Energy Utilization, Zhejiang University, Hangzhou 310003, P. R. China.

J. Chen, Prof. D. A. Weitz

John A. Paulson School of Engineering and Applied Sciences, Harvard University, Cambridge, MA 02138, USA

**Material**

Hyaluronic acid (HA, MW= 10000 g/mol; Shanghai Rhawn Chemical Co.) and Amino-modified silicone oil (NH_2_-PDMS-NH_2_; viscosity: 800-500 mPa·S; Shanghai Macklin Biochemical Technology Co.) are used as the shell. Lauric acid (LA, Sinopharm Chemical Reagent Co.) and stearic acid (SA, Sinopharm Chemical Reagent Co.) are mixed to form eutectic core material. IR-780 iodide (IR780, Sigma-Aldrich.) is used as a photothermal photosensitizer loaded in the nanocapsules. Doxorubicin (DOX, Shanghai Aladdin Biochemical Technology Co.) is a typical lipophilic drug cargo loaded in nanocapsules. Ethanol (Sinopharm Chemical Reagent Co.) is used as the solvent and deionized (DI) water is used as the anti-solvent. Doxorubicin Hydrochloride (DOX HCl, Shanghai Aladdin Biochemical Technology Co.) is used as a contrast group to confirm the toxic side effect of drug.

**Methods**

**Preparation of nanocapsules**

Nanocapsules are prepared by rapid solvent exchange and electrostatic self-assembly. 5mg NH_2_-PDMS-NH_2_, 8 mg LA, 2 mg SA, 5 mg IR780 and 10 mg DOX are co-dissolved in 1 mL ethanol at 55°C. 100 μL ethanol solution is quickly injected into a reservoir of 3 mL 0.5mg/mL HA aqueous solution at 55°C using a 1-200 μL gel-loading pipet tip. Upon solvent exchange, LA, SA, NH_2_-PDMS-NH_2_, IR780, and DOX are co-precipitate into oil cores and HA are sequential self-assemble on the surface of oil cores to form core-shell IR780 and DOX loaded HA nanocapsules (IR780+DOX@NC). The dispersion of nanocapsules in water is then placed in an ice bath to solidify the oil core. Ethanol is removed by evaporation in air and unencapsulated drugs are removed after dialysis in deionized water for 12 h. DOX-loaded HA nanocapsules (DOX@NC), IR780-loaded HA nanocapsules (IR780@NC), and empty HA nanocapsules are prepared following the same procedure. Different concentrations of nanocapsules in water are obtained by dilution.

**Characterization of nanocapsules**

The nanocapsule size and zeta potential are measured by Zetasizer (Nano series; Malvern). Scanning Electron Microscopy (SEM) images are obtained by a scanning electron microscope (SU-70; Hitachi). UV-Vis absorption of DOX and IR780 are measured by a UV-Vis spectrophotometer (UV-1800; Shimadzu). Photothermal effect of IR780 loaded nanocapsule is measured by a K meter (TCM-UA; Dagasensor). Differential scanning calorimetry (DSC, Q200, TA Instruments) is used to test the thermal properties of eutectic oil mixture at different weight ratios (LA: SA = 10:0, 9:1, 8:2, 0:10). The thermal image is obtained by an infrared imager (MLX-90640; Puto Instruments).

**Characterization of encapsulation efficiency and loading capacity**

Encapsulation efficiency of drugs in nanocapsules is determined by the formula, Encapsulation Efficiency = *w*_encapsulated_ _drug_ / *w*_total drug_ × 100%, while loading capacity of drugs in nanocapsules is calculated by the formula, Loading Capacity = *w*_encapsulated drug_ / (*w*_encapsulated drug_ + *w*_nanocapsule_) × 100%, where *w*_encapsulated drug_ is the weight of drugs encapsulated in the nanocapsules, *w*_total drug_ is the total weight of drugs used (*w*_total drug_ = *w*_encapsulated drug_ + *w*_unencapsulated drug_) and *w*_nanocapsule_ is the total weight of nanocapsules (*w*_nanocapsule_ = *w*_oil_ + *w*_polymer_). To determine *w*_unencapsulated drug_, 1 mL of nanocapsules dispersed in water is centrifuged at 16000 rpm for 90 minutes. *w*_unencapsulated drug_ in the supernatant quantified by UV-Vis, while *w*_encapsulated drug_ = *w*_total drug_ - *w*_unencapsulated drug_.

**Photothermal effect of IR780 loaded nanocapsules.**

1mL DI water, 1 mL saturated IR780 water solution and 1 mL of IR780@NC (1mg) dispersed in water are exposed to the 808-nm NIR laser (0.6 W cm-2; MDL-808; LaserTo) for 4 min and temperature is recorded at intervals of 2 s. After temperature drops to ambient temperature, two additional cycles of irradiation are performed with each cycle lasting for 4 min.

**NIR-triggered drug release**

To measure the release profile of nanocapsules under NIR irradiation, 5 mL IR780+DOX@NC dispersed in water is irradiated by an 808 nm NIR laser (0.6 W cm-2) for 20 min. At indicated time points of 5 min, 10 min, 15 min and 20 min, 10 µL solution is taken out and diluted into 1mL solution by DI water. Subsequently, solutions of different time points are centrifuged at 16000 rpm for 90 minutes and then the supernatant is measured by UV-Vis to determine the concentration of released DOX. The percentage of released drugs at a specific time point is calculated by the following formula, Percentage of Released Drug = (*w*_released_ _drug_ / *w*_total drug_) × 100%, where *w*_released_ _drug_ is the weight of released drugs at a specific time point, and *w*_total drug_ is the total weight of drugs used. *w*_released_ _drug_ is determined by UV-Vis measurements. The absorption peak of DOX at λ~488 nm is measured and the concentration of DOX is determined according to its standard absorption curve.

**Cell culture**

Murine hepatoma (Hepa 1-6) cell, derived from the BW7756 hepatoma tumor that arose spontaneously in C57L/J mice, and human liver (HL-7702) cells are obtained from Shanghai Institute for Biological Science. Hepa 1-6 and HL-7702 cells are cultured in DMEM medium (Zhejiang Senrui Biotechnology Corporation) supplemented with 10% fetal bovine serum (FBS) and 1% penicillin-streptomycin and incubated in an incubator with 5% CO_2_ at 37℃.

**Cellular uptake of nanocapsules**

Hepa 1-6 cells are first seeded in a 12-well plate. After incubation for 24 hours, Hepa 1-6 cells are co-cultured with DOX HCL and DOX@NC at a same concentration of 2 μM. After co-culture for 8 hours, Hepa 1-6 cells are washed by PBS three times and then prepared into single-cell suspensions. The cellular uptakes of different nanocapsules by Hepa 1-6 cells are quantitatively measured by flow cytometry (CytoFlex LX; Beckman Coulter). The cellular uptake of HL-7702 cells is estimated following the same procedure. In addition, pharmacokinetics of DOX@NC for different times in Hepa 1-6 cells is evaluated by flow cytometry.

**Biocompatibility and bioavailability of nanocapsules**

The biocompatibility of blank nanocapsules is tested by Hepa 1-6 cells and HL-7702 cells. Hepa 1-6 and HL-7702 cells are seeded in a 96-well plate at a density of 1000 cells/well with 100 μL culture medium. The culture medium is removed after 24 hours’ incubation and replaced by 100 μL fresh culture medium containing different concentrations of blank nanocapsules, e.g. 0 μg/mL, 1 μg/mL, 10 μg/mL, 50 μg/mL, 100 μg/mL and 200 μg/mL. After co-culture for 72 hours, the culture medium is replaced by 100 μL serum-free culture medium and the cell viability is measured by cell counting kit-8 (CCK-8) to evaluate the biocompatibility of blank nanocapsules.

To evaluate the bioavailability of nanocapsules, Hepa 1-6 cells co-cultured with unencapsulated DOX, DOX@NC, IR780@NC and IR780+DOX@NC are irradiated with the 808-nm NIR laser at a power density of 0.6 W cm−2 for different time, including 0, 1, 2.5 and 5 min. In addition, the concentration of DOX in different formulations. After 72 hours’ co-culture, the culture medium is replaced by 100 μL serum-free culture medium and the cell viability of Hepa 1-6 cells in each well is measured by CCK-8.

**Intracellular controlled release characterization of nanocapsules**

Fluorescent confocal microscopy is used to directly observe the cellular uptakes of nanocapsules by Hepa 1-6 cells. Hepa 1-6 cells are first planted on petri dishes. After incubation for 24 hours, Hepa 1-6 cells are co-cultured with IR780+DOX@NC at a same concentration of 5 µg/mL. After co-culture for 24 hours, Hepa 1-6 cells are washed by PBS for three times and then fixed using 4% paraformaldehyde for 15 minutes. The nuclei and lysosome of Hepa 1-6 cells are stained by DAPI (2 μg/mL) and DCHF-DA (2 μg/mL) respectively for 15 minutes. Hepa 1-6 cells are then imaged using a confocal laser scanning microscopy (SP8; Leica). To visualize the intracellular release of nanocapsule, the cells are irradiated with the 808-nm NIR laser at a power density of 0.6 W cm−2 for 10 min, and the fluorescence images are taken at 0, 4, and 8 min.

**Tumor model**

Male C57BL/6 mice of 6 weeks old are purchased from Hangzhou Ziyuan Experimental Animal Technology and housed in the animal facility of the First Affiliated Hospital, Zhejiang University. The procedure and protocol of animal experiments are approved by the Animal Experimental Ethical Inspection of the First Affiliated Hospital, College of Medicine, Zhejiang University (reference number: 2024-1321). 5×10^5^ Hepa 1-6 cells in 100 μL PBS are subcutaneously injected into the right flank of mice. For the local and distant model, 5×10^5^ Hepa 1-6 cells in 25 μL PBS are injected into liver to build an orthotopic tumor model (distant tumor) with a sterile insulin needle.

***In vivo* fluorescent imaging**

To demonstrate the tumor-targeting performance of HA coating nanocapsules, tumor-bearing mice are treated with 200 μL of the dispersion of IR780+DOX@NC through tail vein. Fluorescent images of mice under different treatments are captured using the IVIS Lumina XRMS Series III system (PerkinElmer) at 12 h, 24h, 48h, 72h, 5d and 7d.

Subsequently, the five major organs, i.e. heart, liver, spleen, lung and kidney, are dissected from mice and fluorescence images of the dissected organs are captured by the same imaging system. The fluorescent excitation and emission wavelengths for IR780 are 745 nm and 785 nm, respectively.

***In vivo* drug delivery**

When their tumor volume reaches 50 mm^3^, tumor-bearing mice are randomly divided into four groups: Group 1: PBS (mice treated with PBS), Group 2: IR780@NC with NIR irradiation, Group 3: IR780+DOX@NC without irradiation, and Group 4: IR780+DOX@NC with irradiation. For each group, 200 μL of the corresponding dispersion is injected into mice through tail vein every 2 days for two weeks. Irradiation is performed using an 808 nm NIR laser (1.2 W cm-2) for 20 minutes one day after injection. The DOX concentration in different treatments is kept constant at 10.0 mg/kg. Tumor volume is calculated according to the formula: V = 1/2 × length × width^2^, which is measured every 3 days. All mice are sacrificed after two weeks’ treatment. Tumor samples are collected for histopathologic analysis and fluorescence-activated cell sorting (FACS).

**H&E staining**

To evaluate the inflammation response of nanocapsules, five major organs dissected from mice under different treatments are fixed with 4% paraformaldehyde (P1110; Solarbio) and embedded in paraffin (YA0012; Solarbio). Paraffin-embedded organ samples are cut into 4 μm-thick sections and baked in the oven at 65℃ for 1 hour. The organ samples are then deparaffinized in fresh xylene for 10 minutes for three times and rehydrated subsequently in 100% ethanol, 95% ethanol and 70% ethanol for 5 minutes each. Hydrated organ sections are washed by water and stained by hematoxylin for 10 minutes. The organ samples are then stained with Eosin (E4009; Sigma) for 5 minutes and dehydrated subsequently in 95% ethanol, 100% ethanol and 100% ethanol for 5 minutes each. After dehydration, the organ samples are vitrified in dimethylbenzene for 10 minutes for two times and sealed in neutral balsam. The prepared organ samples are evaluated under an optical microscope.

**4-HNE staining**

Dissected tumor samples are fixed with 4% paraformaldehyde (P1110; Solarbio) and then embedded in paraffin (YA0012; Solarbio). Paraffin-embedded tumor samples are cut into 5 μm-thick sections and baked in the oven at 65℃ for 1 hour. The tumor sections are then deparaffinized in fresh xylene for 10 minutes for three times and rehydrated subsequently in 100% ethanol, 95% ethanol and 70% ethanol for 5 minutes each. Hydrated tumor sections are treated with sodium citrate at 100℃ for 15 minutes for antigen retrieval and then submerged in 3% H2O2 for 10 minutes to quench the activity of endogenous peroxidase. After blocking nonspecific binding sites in 5% BSA for 1 hour, the tumor sections are incubated with 4-hydroxy-2-noneal (4-HNE, 1:400, Abcam, ab46545,) overnight at 4℃. The tumor sections are then incubated with horseradish peroxidase-labelled mouse anti-rabbit secondary antibody for 1 hour at room temperature. Finally, the tumor sections are stained with 3,3’-diaminobenzidine (DAB) for 5 minutes and then stained with hematoxylin for 7 minutes. 4-HNE stained images of the tumor sections are obtained under an optical microscope.

**General ROS quantification**

To measure levels of cellular ROS, cells in 12-well plate after treatments were incubated with fresh medium containing 20 μM H2DCFDF (Invitrogen) per well for 60 min. Then cells were collected and washed once with PBS followed by fluorescence-activated cell sorting (FACS) analysis. Mean fluorescence intensity (MFI) was determined by flow cytometry using FlowJo software.

**Fluorescence-activated cell sorting (FACS)**

Tumor samples dissected from mice under different treatments are digested in 5 mL of culture medium containing 0.01 mg/mL DNase I (11284932001; Merck), 0.1 mg/mL collagenase IV (17104019; Gibco) and 2% FBS for 1 hour at 37°C. Cell suspension is prepared by filtering the digested tumor sample through a 70 µm cell strainer, which is followed by centrifugation at 500 g for 5 minutes and then resuspension in 5 mL of 36% Percoll (P4937; Sigma). After centrifugation at 500 g for 5 minutes, the supernatant is removed and 2 mL blood lysis buffer (555899; BD) is used to lyse the red blood cells for 10 minutes. The sample is then centrifuged at 300 g for 5 minutes and resuspended in 100 μL FC Block (156604; BioLegend) for 15 minutes at 4°C. After centrifugation at 500 g for 5 minutes, the supernatant is removed and the cells are stained with fixable viability stain 780 (565388; BD Biosciences) in 100 μL PBS for 20 minutes at 4°C. Subsequently, after centrifuging at 600 g for 5 minutes and removing the supernatant, the cells are stained by a mixture of antibodies in PBS containing 2% FBS. The antibodies utilized include CD45-BV785 (564225; BD Biosciences), CD3-FITC (100204; BioLegend), CD4-PerCP-Cy5.5 (561115; BD Biosciences), CD8-BV650 (100741; BioLegend), Ki67 (652405; BioLegend), CD44 (560569; BD Biosciences), CD62L (161204; BioLegend) F4/80-PE (123110; BioLegend), CD11b- PerCP-Cy5.5 (101227; BioLegend), Ly6C(128035; BioLegend) and Ly6G (127647; BioLegend), CD11c (117339; BioLegend), MHC-II (107631; BioLegend). Flow cytometry analysis is performed using a flow cytometer (BD LSR Fortessa; BD Bioscience).

**Statistical analysis**

Statistical analyses are performed using GraphPad Prism 8.0 and Origin 2023b. The flow cytometry data are analyzed by FlowJo 10. One-way analysis of variance is used to identify the significant difference between different groups. All data are presented as means ± SEM. Statistically significant differences are indicated as follows: *p < 0.05, **p < 0.01 ***p < 0.001 and ****p < 0.0001.

**Table S1.** Size and zeta potential of nanocapsules with and without cargo.

|  | Size | Zeta potential |
| --- | --- | --- |
| HA nanocapsules | 183.5 ± 44.4 nm | -53.7 ± 5.4 mV |
| IR780+DOX@NC | 179.9 ± 38.5 nm | -55.2 ± 5.2 mV |

**Table S2.** Encapsulation efficiency and drug loading efficiency of different materials loaded in nanocapsules. The total drug could be 5mg DOX, 3mg IR780 or 1mg Nile Red.

|  | Encapsulation efficiency | Drug loading efficiency |
| --- | --- | --- |
| DOX | 81.3 ± 2.4% | 13.55 ± 0.8% |
| IR780 | 89.6 ± 1.6% | 8.96 ± 0.2% |

**Table S3.** At each time point of 5, 10, 15 and 20 mins, the release rate of DOX with/without laser irradiation.

| Time (min) | With the laser irradiation | Without the laser irradiation |
| --- | --- | --- |
| 0 | 0% | 0% |
| 5 | 2.169% | 0.004% |
| 10 | 5.813% | 0.069% |
| 15 | 12.79% | 0.05% |
| 20 | 19.767% | 0.147% |


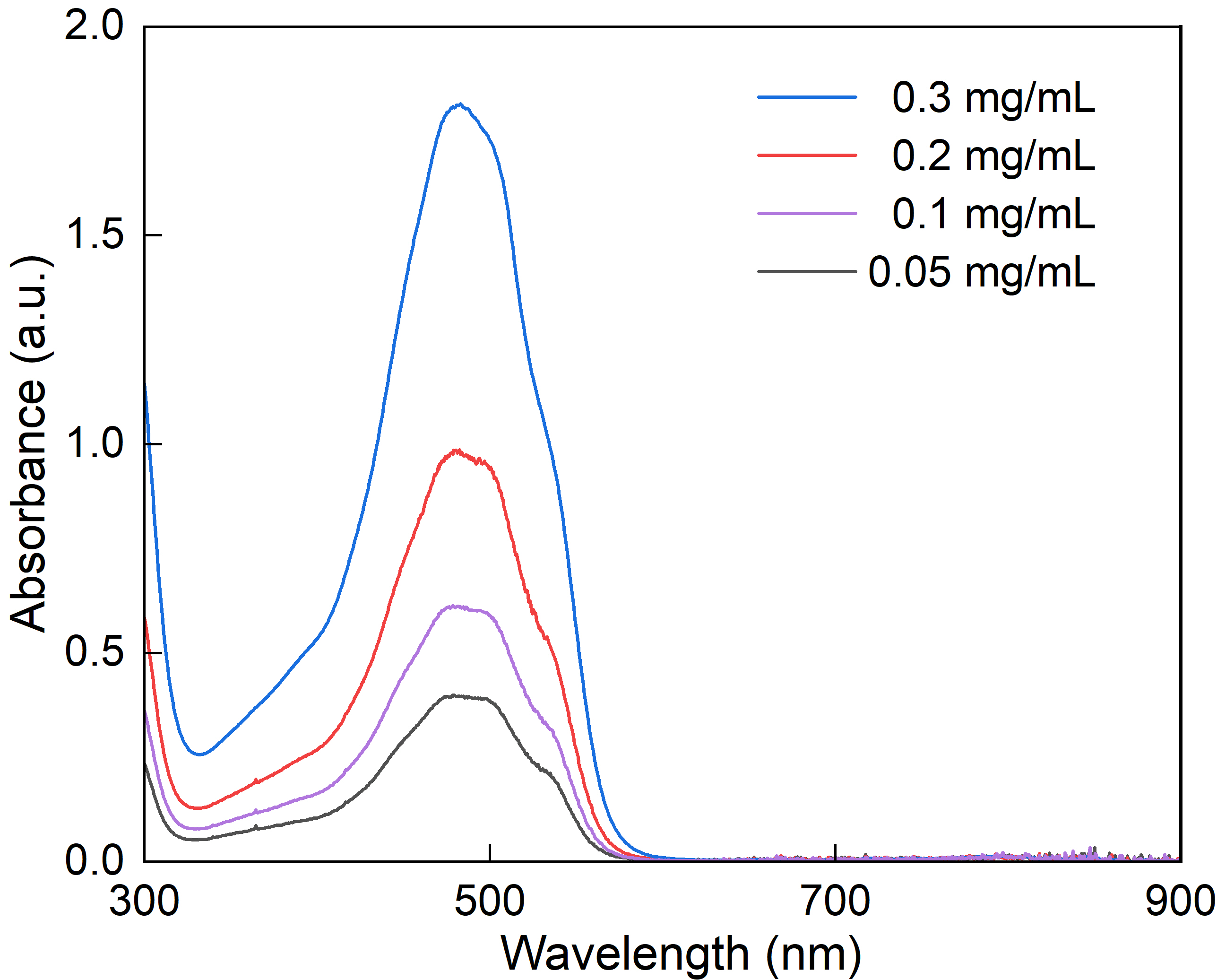


**Figure S1:** UV-Vis spectra of DOX dispersed in water. The UV-Vis absorption of DOX decreases linearly as the dispersion is diluted, which follows the Lambert-Beer law.


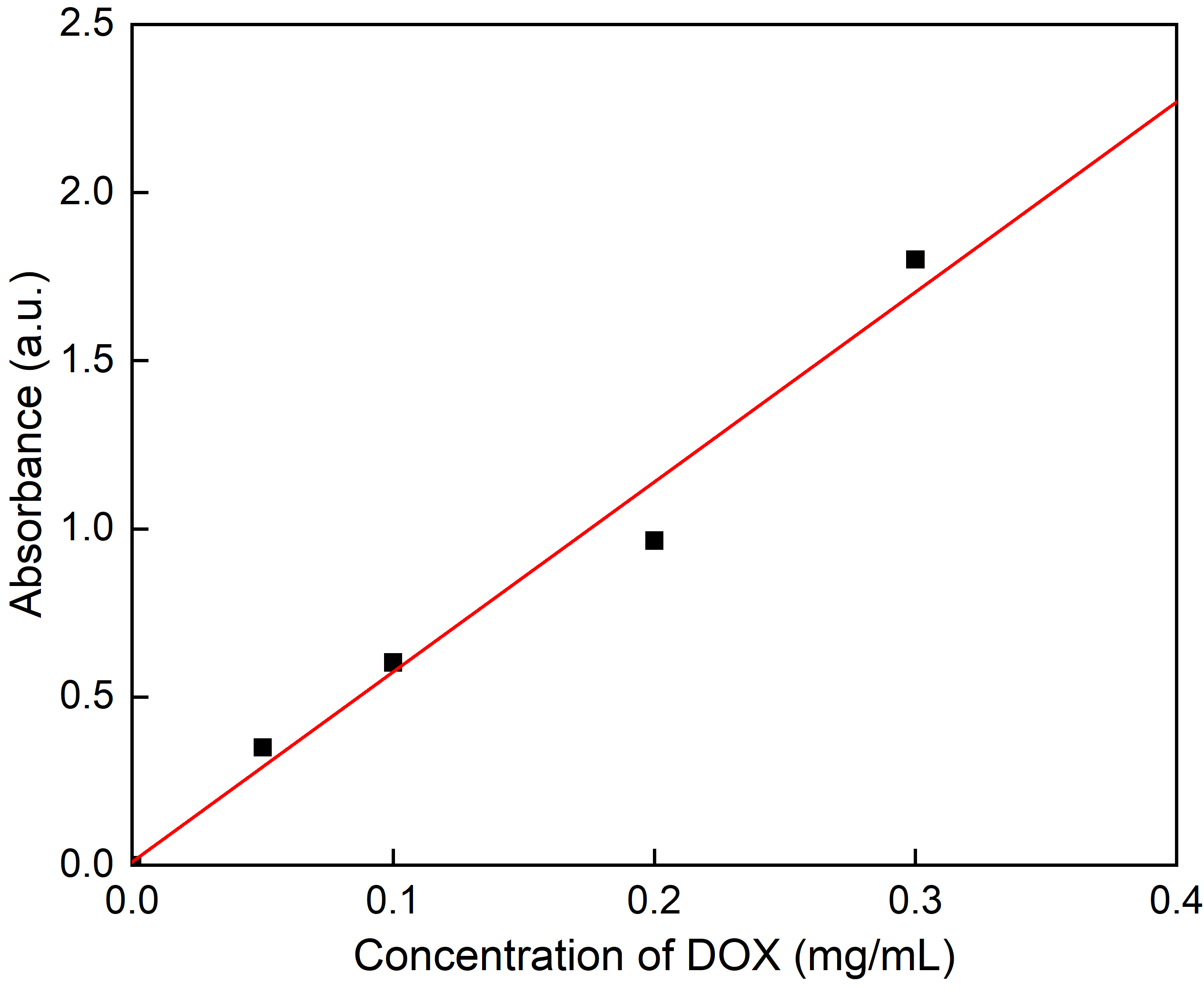


**Figure S2:** Absorption peak of DOX measured at λ~550 nm as a function of DOX concentration, showing a linear dependance.


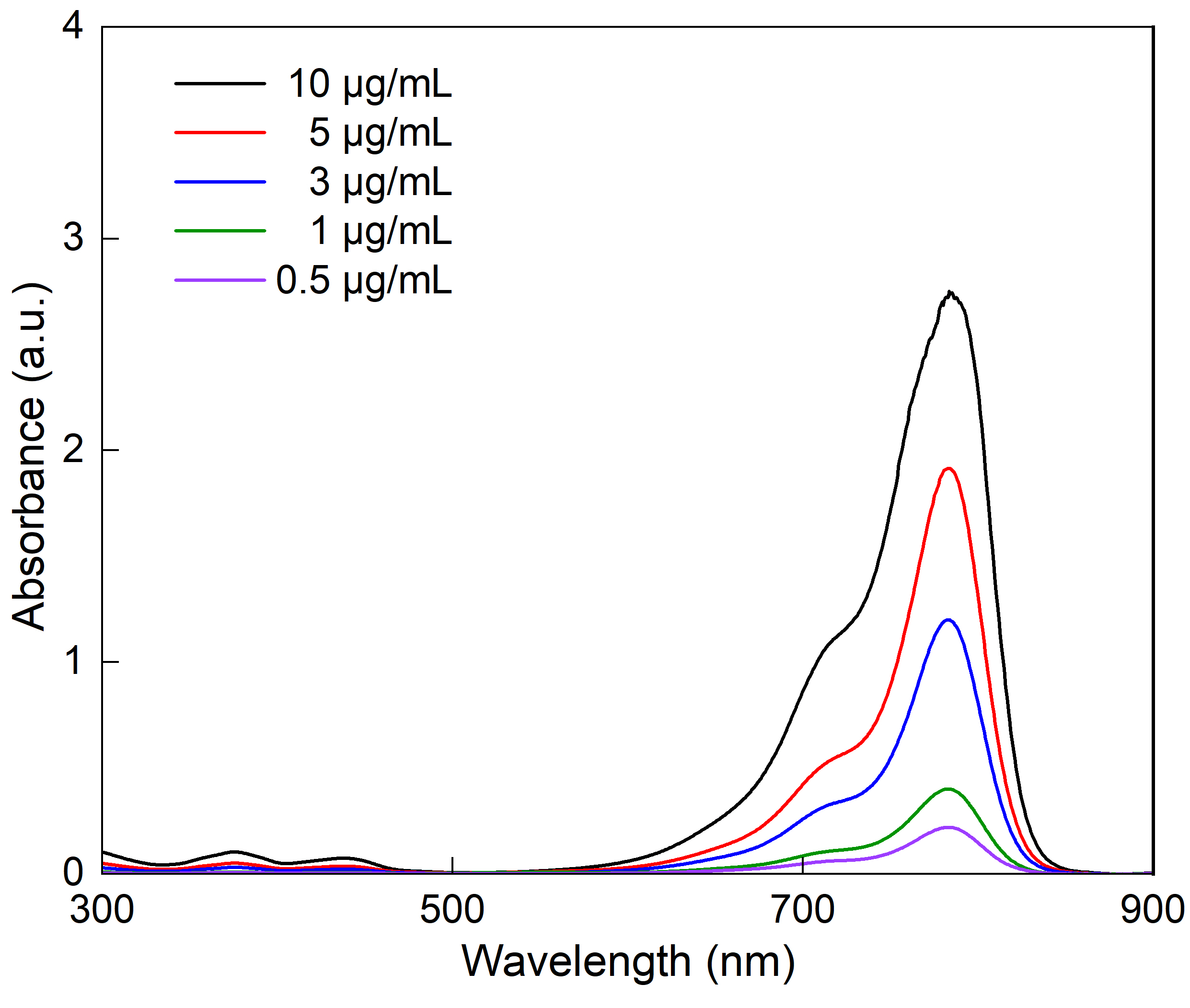


**Figure S3:** UV-Vis spectra of IR780 dispersed in water. The UV-Vis absorption of IR780 decreases linearly as the dispersion is diluted, which follows the Lambert-Beer law.


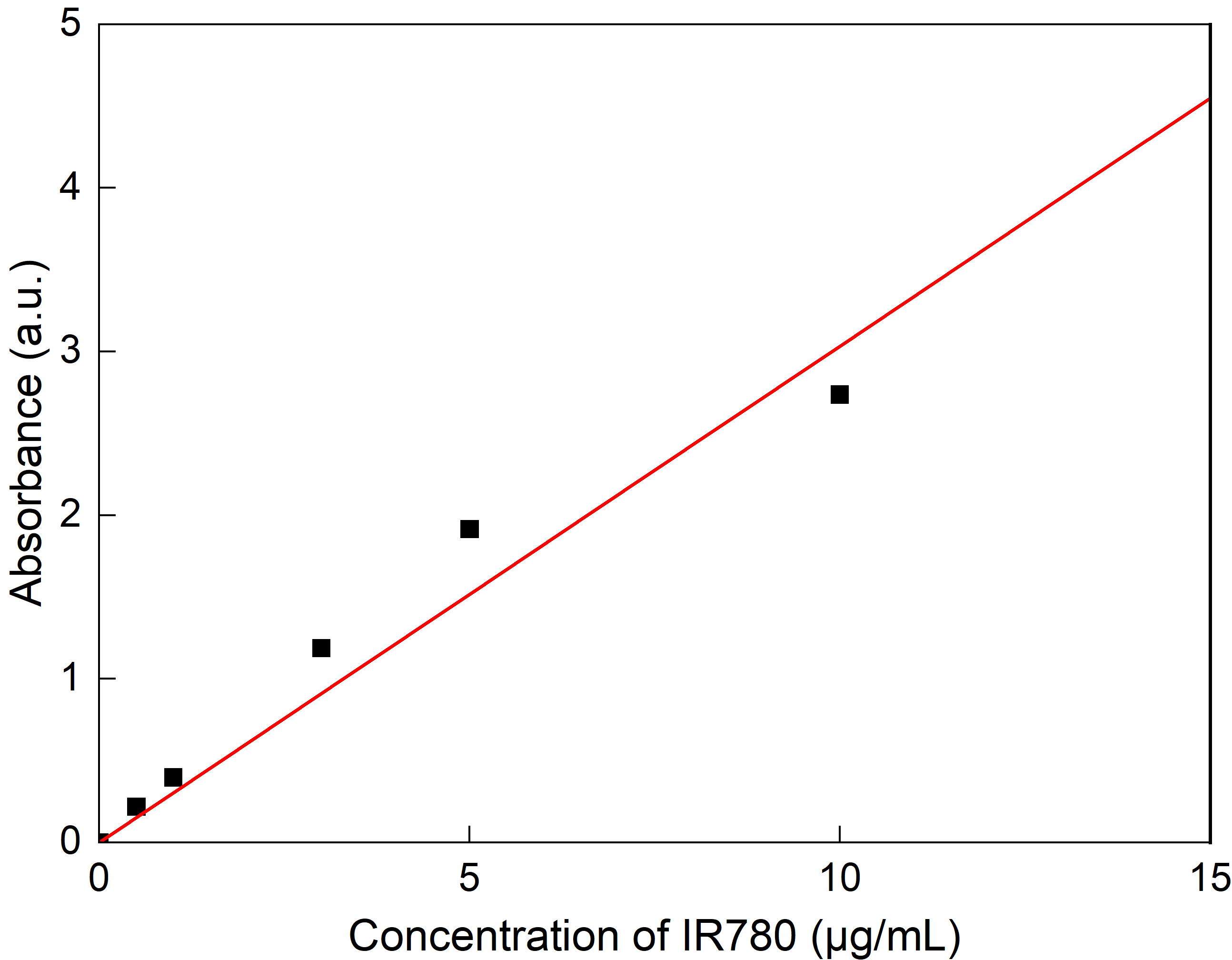


**Figure S4:** Absorption peak of IR780 measured at λ~550 nm as a function of IR780 concentration, showing a linear dependance.

**Figure S5:** Stability of IR780@NC and free IR780 disperse in water after 3h. The IR780 loaded in nanocapsules show a great resistance of degradation.


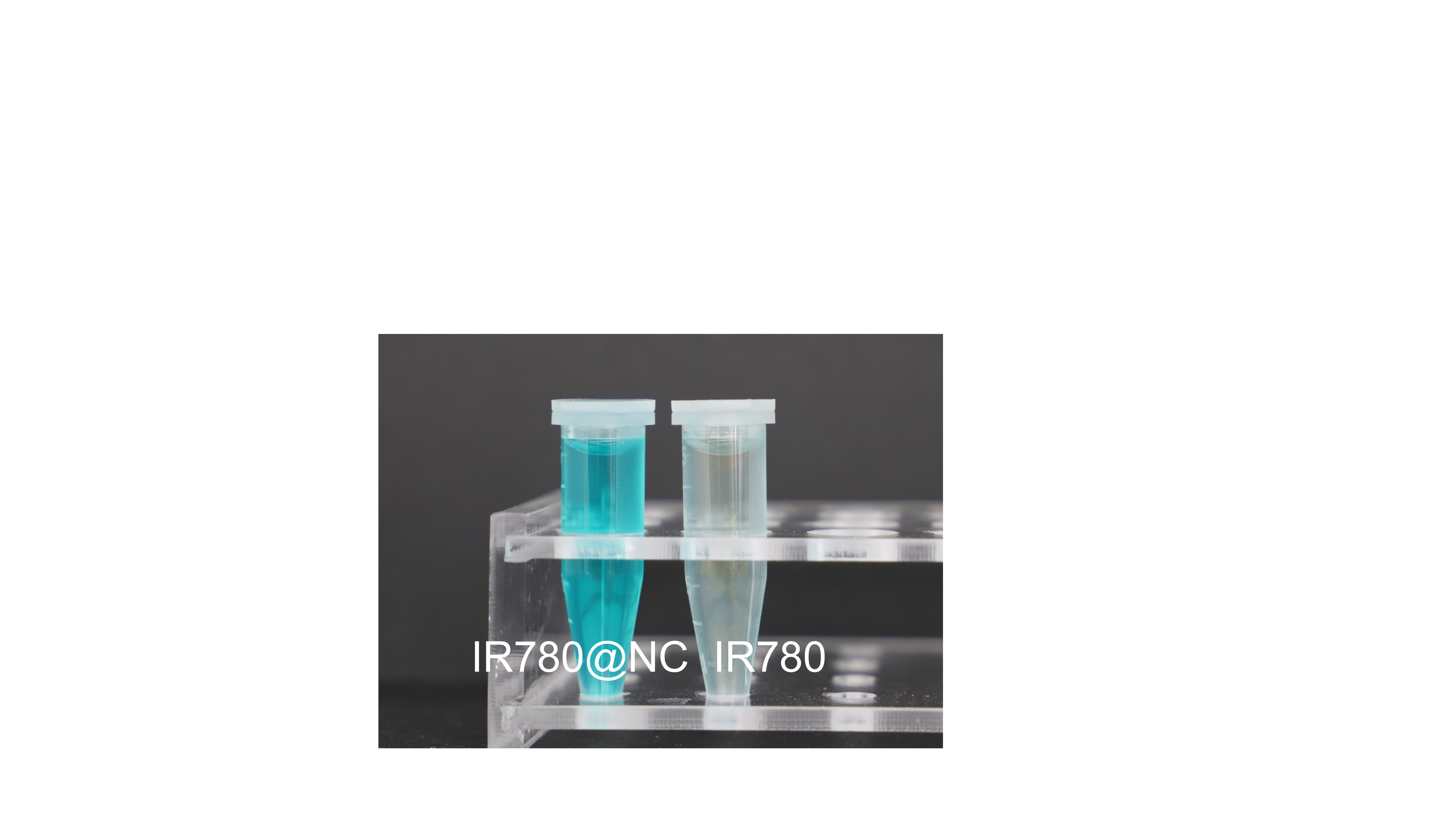

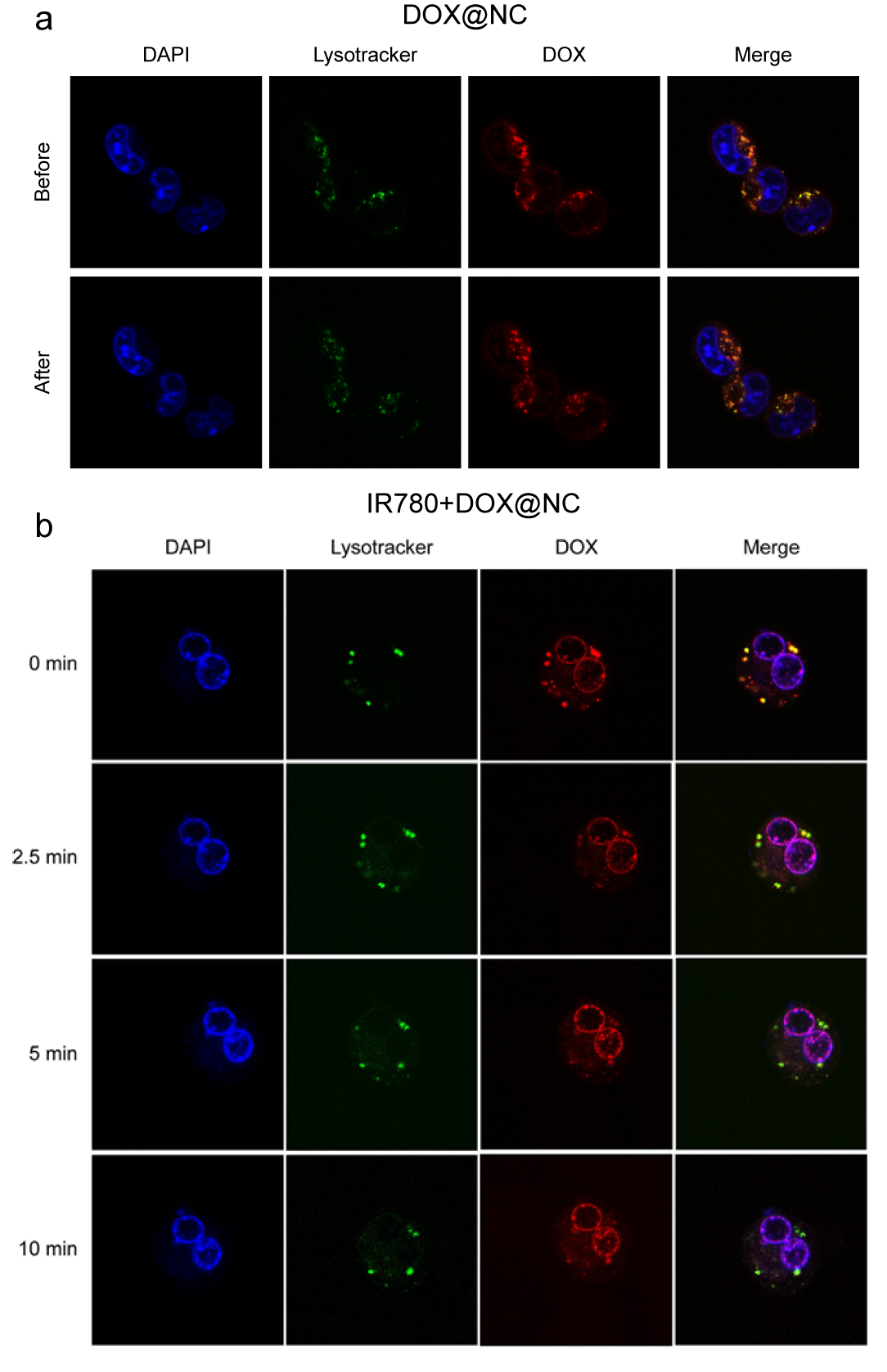


**Figure S6.** (a) Cellular uptake of the nanocapsules and demonstration of NIR-triggered drug release. Fluorescence images showing the locations of nuclear, lysotracker and DOX in Heap 1-6 cells in response to the 808 nm NIR laser at a power density of 0.6 W cm−2. (b) Cellular uptake of the DOX@NC nanocapsules


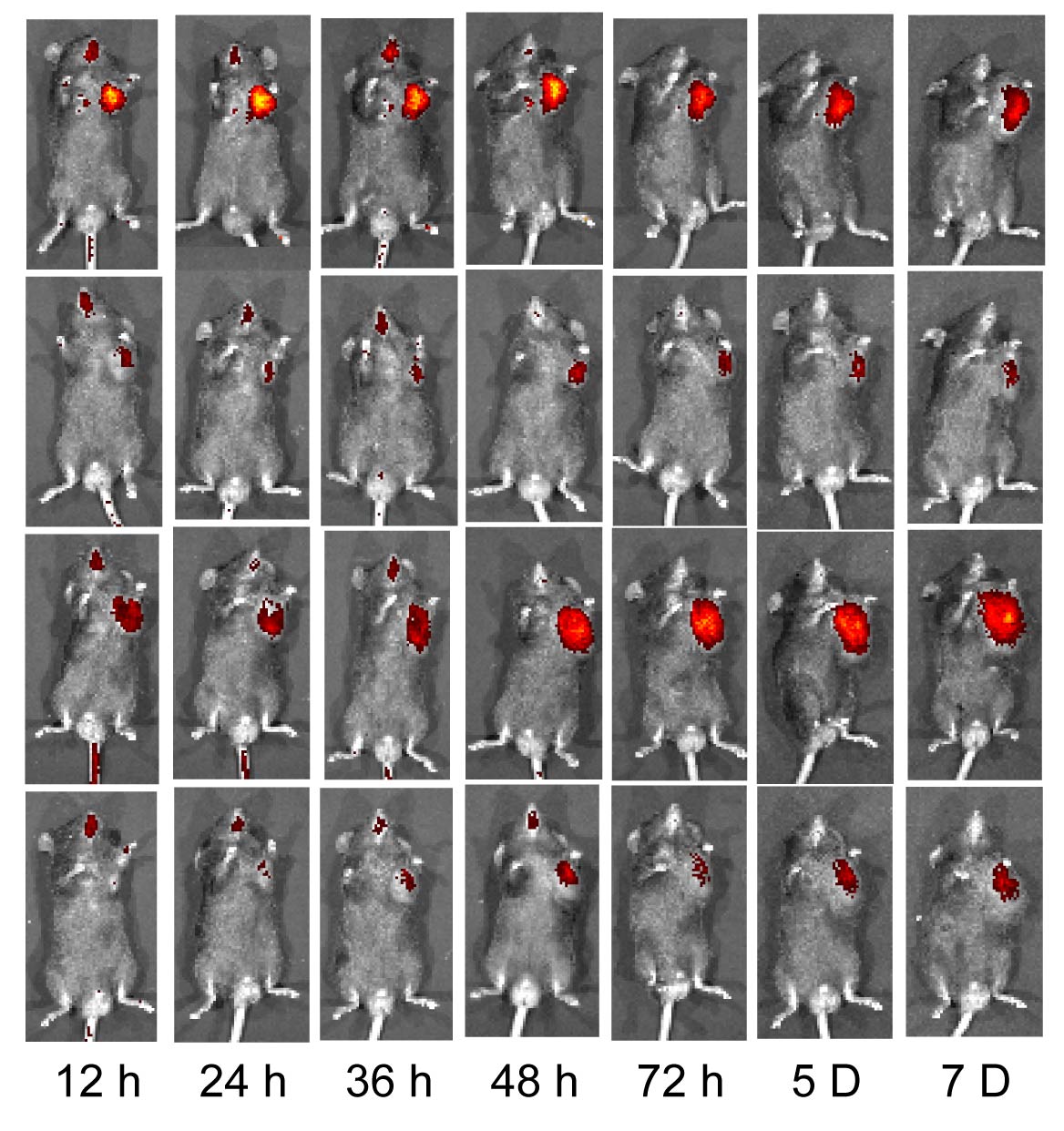


**Figure S7:** *In vivo* imaging of mice after intravenous injection of1 IR780@NC.


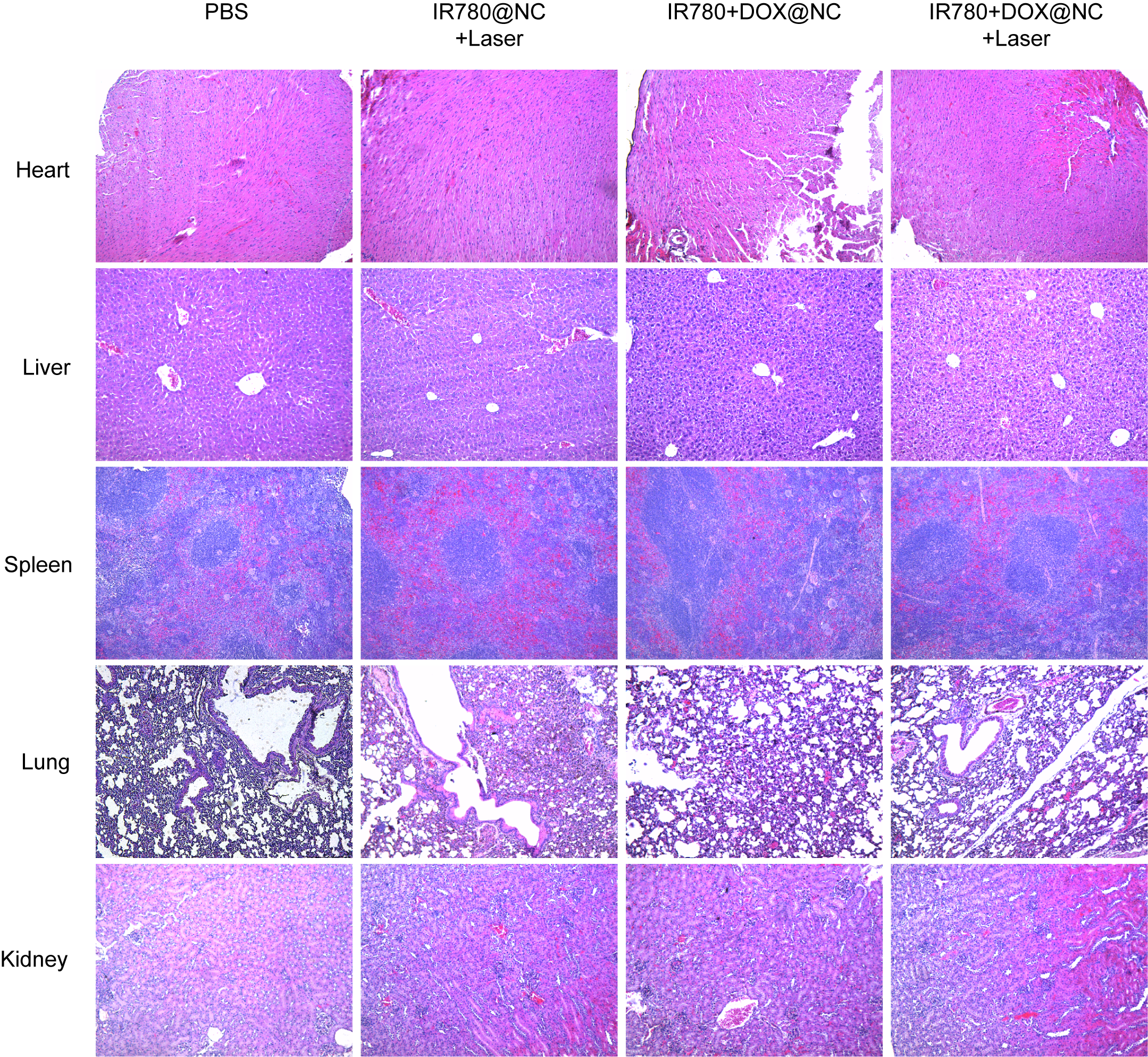


**Figure S8:** Safety evaluation of drug-loaded nanocapsules. H&E-stained images of heart, liver, spleen, lung and kidney from mice treated with PBS, IR780@NC with irradiation, IR780+DOX@NC without irradiation, and IR780+DOX@NC with irradiation. No inflammation response is observed in the five major organs, suggesting a low off-site effect and a good safety of drug-loaded nanocapsules, which is attributed to their good biocompatibility and tumor-targeting capability.


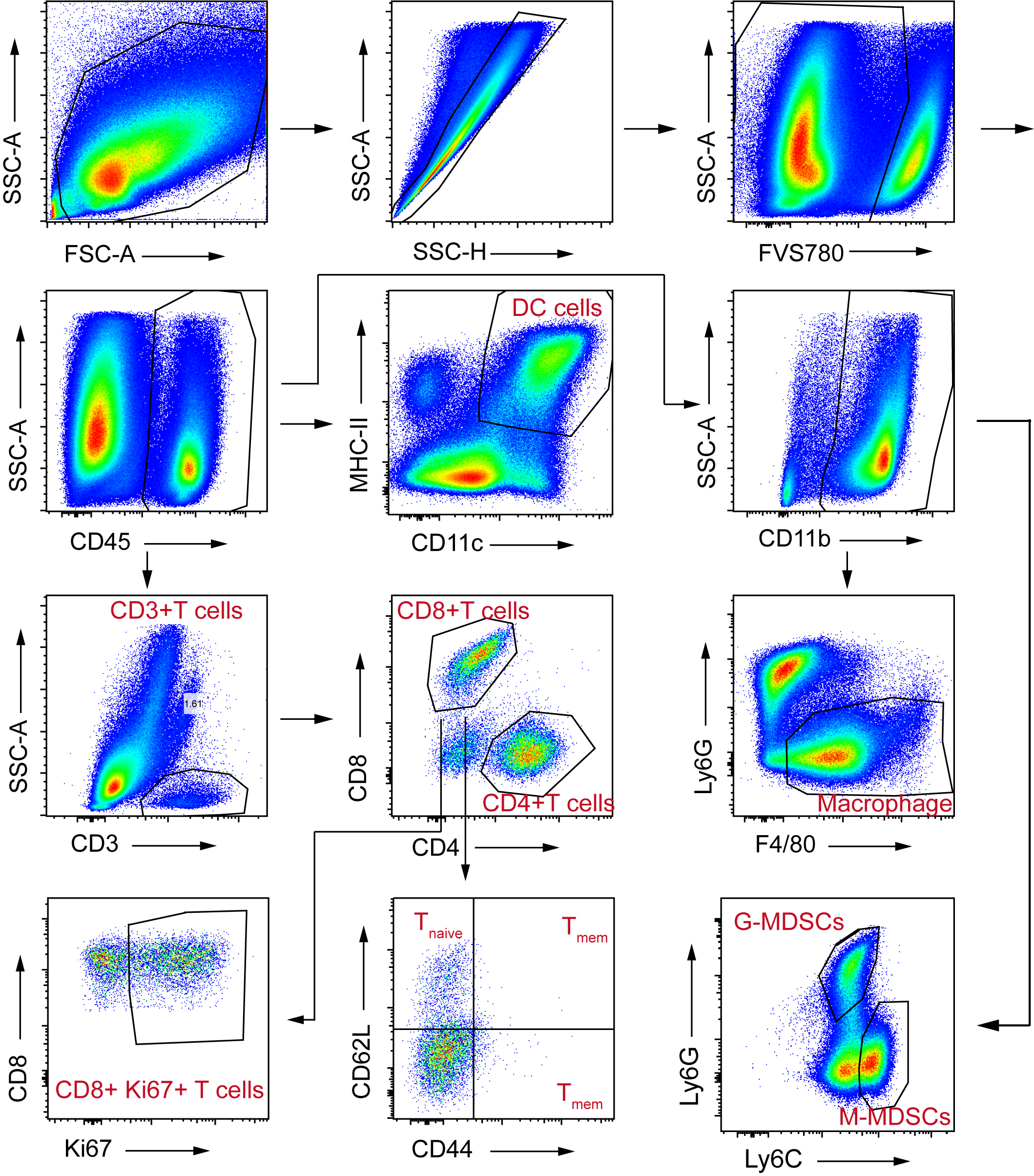


**Figure S9:** Gating strategy for analyzing the ratio of cytotoxic T lymphocytes (CTLs) by flow cytometry.
